# Magnesium sulfate for postoperative pain in orthopedic surgery: A narrative review

**DOI:** 10.1097/MD.0000000000038522

**Published:** 2024-06-14

**Authors:** Lana Sbitan, Ayman Issa Nabhan, Bana Zuhair Alafandi, Omar Alzraikat, Noor Alzraikat

**Affiliations:** a Faculty of Medicine, The Hashemite University, Zarqa, Jordan; b Faculty of Medicine, Al Andalus University for Medical Sciences, Tartus, Syria; c Faculty of Medicine, University of Aleppo, Aleppo, Syria; d Faculty of Medicine, Jordan University of Science and Technology, Irbid, Jordan; e King Hussein Medical Center, Royal Medical Services, Amman, Jordan.

**Keywords:** analgesia, anesthesia, intrathecal MgSO4, intravenous MgSO4, orthopedics, postoperative pain

## Abstract

Magnesium Sulfate (MgSO4) is a widely used adjuvant in anesthesia. Often administered with local anesthetics, it is known to reduce analgesic and opioid consumption while extending the duration of analgesia. MgSO4 applications extend to orthopedic surgeries, cardiovascular and urogenital procedures, offering extended postoperative pain relief. While commonly administered through various routes, there is a research gap concerning the comparative efficacy of intrathecal (IT) and intravenous (IV) MgSO4 administration. This narrative review aims to provide a comparison between IT and IV administration of MgSO4 particularly following orthopedic procedures, where pain management is paramount. A comprehensive literature search was conducted across several electronic databases, trial registries, and gray literature from inception to 2023. Inclusion criteria encompassed studies investigating the effects of perioperative IT administration of magnesium compared to perioperative IV administration of MgSO4 in patients undergoing surgery, with no language restrictions. Our search identified 4326 articles, of which 9 randomized controlled trials met our inclusion criteria. We summarized these selected articles. Four studies discussed IT magnesium sulfate (MgSO4) administration, 2 focused on IT administration in orthopedic surgeries, and 3 explored both IV and IT administration of MgSO4 in orthopedic surgery. IT MgSO4 shows promise in postoperative pain management, delaying block onset and extending duration. Personalized administration choice, considering patient factors and surgery type, is crucial. Further research is needed to refine strategies for better patient outcomes, particularly following orthopedic surgeries.

## 1. Introduction

Magnesium Sulfate (MgSO4) is among the 5 most used adjuvants in anesthesia,^[1]^ often given as an adjuvant with a local anesthetic such as bupivacaine to reduce analgesic consumption, prolong analgesic time,^[2]^ and reduce opioid use.^[3]^ The use of MgSO4 can be attributed to its use in specific specialties or it can be due to the recent attention multimodal anesthetic techniques have garnered.^[1]^ Published guidelines recommend the use of opioid-free analgesia and multimodal treatment methods to relieve postoperative pain.^[2]^

MgSO4 works by antagonizing N-methyl-D-aspartate (NMDA) receptors in spinal neurons, leading to the attenuation of central and peripheral sensitization, ultimately decreasing the risk of chronic persistent postoperative pain after operations such as total knee arthroplasty.^[2,4]^ Furthermore, MgSO4 inhibits calcium influx into cells via voltage-gated channels, which reduces neuronal excitability and causes an anti-nociceptive effect for acute pain.^[3,5]^ Additionally, it can increase the clinical duration of neuromuscular blockers, thus reducing the frequency of their use.^[1]^

MgSO4 has anti-inflammatory and anti-modulatory effects and strengthens chondral regeneration.^[2]^ Other benefits include prevention and treatment of preeclampsia and eclampsia along with many other uses in intensive care or emergencies.^[1]^ While generally safe at recommended doses, MgSO4 can lead to hypotension, residual neuromuscular blockade, hypermagnesemia, intravenous (IV) injection pain, and respiratory depression.^[1]^

MgSO4 has been observed to decrease analgesic consumption and increase postoperative analgesia in urogenital, orthopedic, and cardiovascular surgeries. The use of MgSO4 has been shown to decrease extubation time in cardiovascular surgeries. A decrease in mean arterial pressure was also observed in those who underwent gastrointestinal, orthopedic, and urogenital surgeries. The duration of postoperative analgesia is highest in orthopedic surgeries. The benefits of MgSO4 start to appear in the first 24 hours following orthopedic and cardiovascular surgeries, and the pain relief lasts for 48 hours after orthopedic surgeries. Conversely, the analgesic effect of MgSO4 starts to weaken only 24 hours after urogenital and cardiovascular surgeries.^[6]^

In total knee arthroplasties, MgSO4 given with spinal anesthesia has been found to decrease the incidence of chronic persistent postoperative pain 1 year after the surgery, improve postoperative analgesia, and prolong the duration of the spinal sensorial block.^[3]^

For arthroscopic surgeries, it is essential to use multimodal analgesic methods to provide sufficient pain relief for early mobilization and rehabilitation. Local anesthetics on their own have some limitations including shorter duration of action, low availability in wards, chondrotoxicity, and strict monitoring for side effects. So, it is optimal to combine it with MgSO4 to prolong postoperative analgesia, decrease the dose of analgesics, and protect chondrocytes.^[7]^

In the recent era, the use of intrathecal (IT) adjuvants has gained traction due to its ability to extend the duration of the block, improve patient well-being, and decrease resource utilization compared with general anesthesia.^[8]^

Currently, there is a gap in the literature regarding the use of IT MgSO4 in orthopedic surgery compared with the IV route of administration. This study aims to help bridge this gap by discussing the evidence related to IT use in orthopedics and providing extensive comparisons between the 2 routes.

## 2. Methods

### 2.1. Search strategy and inclusion criteria

From inception up until May 2023, a comprehensive search was conducted across various databases including Medline, Embase, Lilacs, Web of Science, EBSCO, and Scopus, as well as trial registries such as the ISRCTN registry, ClinicalTrials.gov, the Australian New Zealand Clinical Trials Registry, the World Health Organization International Clinical Trials Registry Platform (ICTRP), and the EU Clinical Trials Register. Additionally, a thorough search was performed within The Journal of Negative Results in BioMedicine and gray literature sources. The search strategy employed the following keywords: Orthopedics, IT MgSO4, IV MgSO4. We included studies investigating the effects of perioperative IT administration of MgSO4 compared to other interventions in patients undergoing surgery of all ages. Our focus was on outcomes including the time of onset of sensory and motor blockade, the time of regression of sensory and motor blockade, as well as the duration of anesthesia. We did not use any language restrictions and translated any non-English articles.

### 2.2. Study selection

Two independent reviewers (A.I.N and B.Z.A) screened the articles based on title, abstract, and full text. Each reviewer examined half of the studies. Any conflict in the selection was reviewed by a third reviewer (L.S.), and a consensus was established between all 3 reviewers to resolve any conflict. Upon the completion of the thorough screening process using the predetermined criteria, these selected articles were then methodically tabulated and summarized to provide a coherent guide for the authors.

Since this is a narrative review, there was no need for ethical approval.

### 2.3. Quality assessment

We employed the cochrane risk-of-bias tool for randomized trials (RoB v2) to conduct a quality assessment of the included studies.^[9]^ This tool evaluated the randomization process, deviations from intended interventions, missing outcome data, outcome measurement, and selection of the reported results.

## 3. Results

### 3.1. Study characteristics

**Figure 1.**
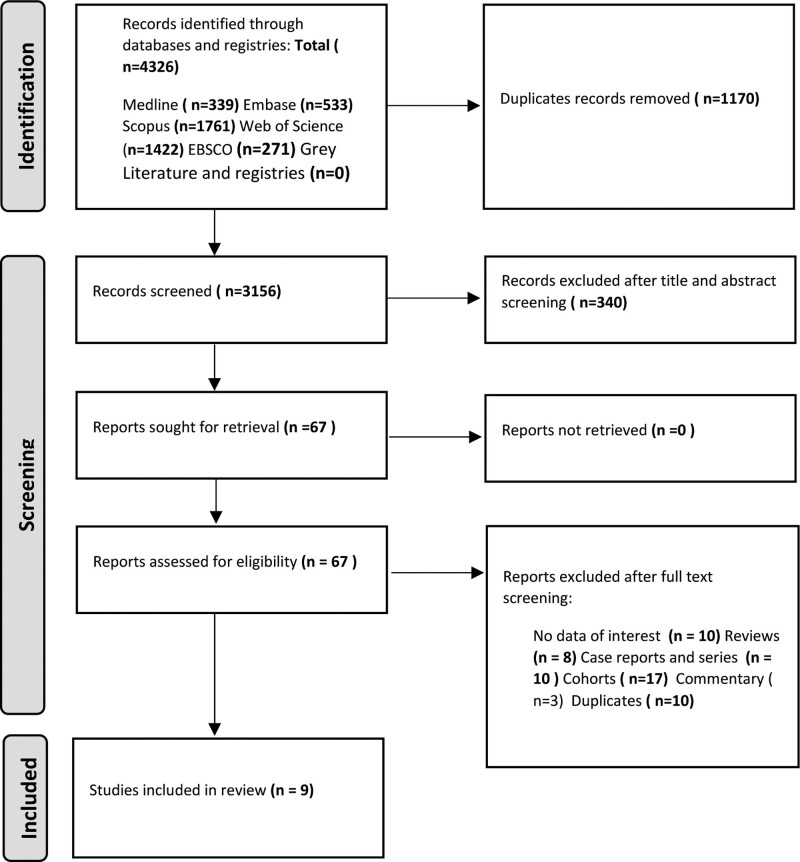
Flow diagram summarizing study selection process.

Following this approach, we conducted a comprehensive evaluation of the IT administration of MgSO4 in orthopedic surgery. A total of 9 randomized controlled trials were deemed suitable for inclusion in our review. These selected articles were meticulously summarized to offer a clear and concise overview of the pertinent findings. Among the included articles, 4 discussed the IT administration of MgSO4, 2 focused on the IT administration of MgSO4 in orthopedic surgeries, and 3 explored the effects of both IV and IT administration of MgSO4 in orthopedic surgery (refer to Fig. 1, representing a flow chart describing the structure of the review).

### 3.2. Quality assessment

Using the Cochrane Quality Assessment Tool (RoB V2),^[9]^ the quality assessment results for the included randomized clinical trials are provided in the Supplemental Digital Content, http://links.lww.com/MD/M798. Seven studies demonstrated an overall low risk-of-bias,^[8,10–15]^ while one study was categorized as having a high risk-of-bias,^[16]^ and one study had an unclear risk.^[17]^

In terms of randomization, all included studies provided details about the randomization process and were categorized as having a low risk in this domain.^[8,10–17]^ Regarding deviations from intended interventions, only 2 studies had an unclear risk-of-bias.^[16,17]^ Notably, one study exhibited a high risk-of-bias in the domain of missing outcome data.^[16]^

Concerning the measurement of the outcome, Four studies were assessed as having an unclear risk-of-bias.^[12,14–16]^ Finally, for the selection of reported results, one study was categorized as having a high risk,^[16]^ and another had an unclear risk.^[17]^

## 4. Discussion

### 4.1. IT MgSO4 use in surgery

The analgesic properties of magnesium are attributed to its impact on NMDA receptors as it hinders central sensitization by blocking excessive activation of pathways in the dorsal horn of the spinal cord. However, magnesium has a restricted ability to penetrate the blood-brain barrier thereby limiting its analgesic effects, as well as the adverse side effects of elevated magnesium serum levels. Therefore, the IT administration of magnesium can help avoid these side effects and potentiate opiates within the cerebrospinal fluid (CSF) during spinal analgesia.^[18]^

IT MgSO4 has been widely used as an adjuvant to local anesthetics in spinal anesthesia. Looking into the literature, we included 4 studies that reported the effects of the administration of IT MgSO4 in spinal anesthesia compared to different adjuvants added to hyperbaric bupivacaine.^[8,10–12]^ Adult patients of American Society of Anesthesiologists (ASA) physical status I and II of either gender undergoing lower abdominal or lower limb surgeries were included in these studies,^[8,10,12]^ except for one study on pregnant women undergoing cesarean section.^[11]^ Patients who were allergic to the study drugs, obese, and suffered from uncontrolled hypertension or any contraindication to spinal anesthesia were mainly excluded.

IT MgSO4 was compared to Nalbuphine,^[10]^ Dexmedetomidine,^[8]^ Sodium Chloride,^[11]^ and Normal Saline,^[8,12]^ s an adjuvant to bupivacaine. Details regarding the baseline characteristics of patients included in these studies are further demonstrated in Table 1.

**Table 1:** Summary of clinical studies using intrathecal magnesium sulfate.

Study ID	Country	Type of surgery	Intervention	Control	Conclusion
Shukla et al 2011^[1]^	India	Lower abdominal and Lower limb	- M group:15 mg hyperbaric bupivacaine + 0.1 mL (50 mg) magnesium sulfate (n^a^^ ^= 30)	- D group: 15 mg hyperbaric bupivacaine + 0.1 mL (10 μg) dexmedetomidine (n = 30) - C group: 15 mg hyperbaric bupivacaine + 0.1 mL saline (n = 30)	Magnesium sulfate group (M): - Delayed onset of block - Extended duration of anesthesia compared to the control group (C), but not as much as the dexmedetomidine group (D) Dexmedetomidine group (D): - Fast and long-lasting onset of anesthesia Both groups: - Similar results in hemodynamic variables - No significant side effects were observed in either group
Choudhury et al 2016^[2]^	India	Lower abdominal	(100 mg/0.2 mL) 50% preservative-free magnesium sulfate along with 3 mL of 0.5% Bupivacaine to a total volume of 3.2 mL (n = 50)	(0.8 mg/0.2 mL) 0.4% Nalbuphine along with 3 mL of 0.5% Bupivacaine to a total volume of 3.2 mL (n = 50)	Nalbuphine as an adjuvant with bupivacaine in spinal anesthesia: - Rapid onset of anesthesia - Prolonged postoperative analgesia Comparison with magnesium sulfate: - Faster onset of anesthesia - Longer postoperative analgesia
Xiao et al 2017^[3]^	China	Cesarean section	A mixed solution of: 0.5% bupivacaine + sufentanil 5 μg + 0.5 mL 10% dextrose + 0.1 mL 50% preservative-free magnesium sulfate (50 mg) diluted with 0.9% sodium chloride to a total volume of 3 mL (n = 30)	A mixed solution of: 0.5% bupivacaine + sufentanil 5 μg + 0.5 mL 10% dextrose, diluted with 0.9% sodium chloride to a total volume of 3 mL (n = 30)	Inclusion of intrathecal magnesium sulfate (50 mg) in cesarean delivery patients receiving spinal anesthesia: - Did not decrease the median effective dose (ED50) of intrathecal bupivacaine - Extended duration of spinal anesthesia - Reduced need for postoperative fentanyl - Delayed onset of sensory and motor blockade associated with spinal anesthesia No significant additional side effects observed
Sen et al 2020^[4]^	India	Infraumbilical	12.5 mg of hyperbaric bupivacaine + 25 mcg of fentanyl + 50 mg of magnesium sulfate to total volume of 4 mL (n = 35)	12.5 mg of hyperbaric bupivacaine + 25 mcg of fentanyl + 1 mL of normal saline to total volume of 4 mL (n = 35)	Addition of 50 mg intrathecal magnesium sulfate to subarachnoid block: - Prolongs onset and duration of analgesia - Extends onset and recovery from motor block No significant changes in hemodynamic variables No adverse effects reported

a n: number of patients.

1. Shukla D, Verma A, Agarwal A, Pandey HD, Tyagi C. Comparative study of intrathecal dexmedetomidine with intrathecal magnesium sulfate used as adjuvants to bupivacaine. *J Anaesthesiol Clin Pharmacol*. 2011;27(4):495-499. doi:10.4103/0970-9185.86594

2. Choudhury B, Pathak D, Chauhan RC, L C, Mondal D. Effect of intrathecal nalbuphine and magnesium sulfate used as adjuvants with bupivacaine in spinal anesthesia for lower abdominal surgery: a comparison. *J Evol Med Dent Sci*. 2016;5:4922-4926. doi:10.14260/jemds/2016/1118

3. Xiao F, Xu W, Feng Y, et al Intrathecal magnesium sulfate does not reduce the ED50 of intrathecal hyperbaric bupivacaine for cesarean delivery in healthy parturients: a prospective, double blinded, randomized dose-response trial using the sequential allocation method. *BMC Anesthesiol*. 2017;17:8. doi:10.1186/s12871-017-0300-z

4. Sen J, Singh S, Sen B. The effect of intrathecal magnesium sulfate on bupivacaine-fentanyl subarachnoid block for infraumbilical surgeries. *J Evol Med Dent Sci*. 2020;9(10):780-785. doi:10.14260/jemds/2020/170

The onset time for sensory and motor block, time to sensory and motor regression, and duration of anesthesia were studied among the different groups. The onset of motor block was assessed at a Bromage grade 3 level, and the onset of sensory block was recorded at the T10 dermatome level. However, the T8 dermatome level was accepted by Sen et al as the onset of sensory block,^[12]^ and the time of a Bromage score of 1 was recorded as motor block onset by Xiao et al.^[11]^

All studies reported significantly delayed onset of both sensory and motor block in the MgSO4 groups compared to other adjuvants. Additionally, dexmedetomidine was found superior to both MgSO4 and normal saline in terms of rapid onset of sensory and motor blockade.^[8]^ The sensory and motor block regression time was significantly prolonged in the MgSO4 groups, but the dexmedetomidine group showed a slightly longer regression time.

Xiao et al studied the effect of IT MgSO4 on reducing the median effective dose (ED50) of IT hyperbaric bupivacaine in spinal bupivacaine-sufentanil anesthesia for cesarean section and found that the addition of MgSO4 did not reduce the ED50, but prolonged the duration of effective anesthesia,^[11]^ which is inconsistent with another study on patients undergoing infraumbilical surgeries showing that IT MgSO4, when added to hyperbaric bupivacaine for spinal anesthesia, significantly prolongs the duration of anesthesia.^[12]^ Meanwhile, Choudhury et al reported a significantly shorter duration of effective anesthesia recorded in the IT MgSO4 group compared to the Nalbuphine group.^[10]^

Only 2 studies reported the adverse side effects of IT MgSO4 in spinal anesthesia, including hypotension, bradycardia, nausea, and vomiting. However, they were all found statistically insignificant.^[10,11]^

Accordingly, IT MgSO4 as an adjuvant to hyperbaric bupivacaine in spinal anesthesia for lower abdomen and lower limb surgeries, as well as cesarean sections, significantly prolongs the duration of effective anesthesia and delays sensory and motor block regression. Whereas Nalbuphine and Dexmedetomidine had a faster onset of anesthesia.

### 4.2. IT MgSO4 use in orthopedic surgery

Upon review of the literature for relevant studies, we found 2 papers addressing the use of MgSO4 for spinal anesthesia in orthopedic surgery.^[13,14]^ Comparators included neostigmine^[13]^ and placebo.^[13,14]^ The main inclusion criteria were eligibility for lower limb surgery; while the main exclusion criteria were contraindication for spinal anesthesia and coexisting comorbidities Table 2.

**Table 2:** Summary of clinical studies using intrathecal magnesium sulfate in orthopedic surgeries.

Study ID	Country	Type of Surgery	Intervention	Control	Conclusion
Faiz et al 2012^[1]^	Iran	Orthopedic lower limb surgery	- Group B: Bupivacaine plus magnesium sulfate 50% (n^a^ = 30)	- Group A: bupivacaine 0.5% Spinal heavy 4 cc. (n = 30) - Group C: bupivacaine plus neostigmine 0.5 mg/mL. (n = 30)	Adding magnesium sulfate: - Efficiently increases motor block onset time - Enhances the safety of the procedure
Kathuria at al. 2014^[2]^	India	Orthopedic lower limb surgery	- Group II: received bupivacaine (0.5%), 12.5 mg + 0.2 mL (50 mg) of preservative-free 25 % magnesium sulfate + 0.3 mL of preservative-free 0.9% normal saline (n = 30) - Group III: received bupivacaine (0.5%) 12.5 mg + 0.3 mL (75 mg) of 25 % magnesium sulfate + 0.2 mL of preservative-free 0.9% normal saline for subarachnoid block (n = 30)	- Group I: received bupivacaine (0.5%), 12.5 mg + 0.5 mL of preservative-free 0.9% normal saline. (n = 30)	Intrathecal delivery of magnesium combined with local anesthesia mixtures: - Increases spinal anesthesia duration - Does not increase adverse events

a n: number of patients.

1.Faiz SHR, Rahimzadeh P, Sakhaei M, Imani F, Derakhshan P. Anesthetic effects of adding intrathecal neostigmine or magnesium sulfate to bupivacaine in patients under lower extremities surgeries. *J Res Med Sci Off J Isfahan Univ Med Sci*. 2012;17(10):918-922.

2.Kathuria B, Luthra N, Gupta A, Grewal A, Sood D. Comparative efficacy of two different dosages of intrathecal magnesium sulfate supplementation in subarachnoid block. *J Clin Diagn Res JCDR*. 2014;8(6):GC01-GC05. doi:10.7860/JCDR/2014/8295.4510

The most frequently reported primary outcomes were sensory and motor block onset and recovery, as well as duration of analgesia. MgSO4 was shown to be a good addition to the anesthesia mixture regarding the duration of sensory and motor block. Comparing magnesium to placebo and neostigmine contributes to a better understanding of magnesium effect on anesthesia parameters and side effects.

#### 4.2.1. Comparison with placebo

Kathuria study showed a significant prolonged onset of sensory block in magnesium groups, while Faiz study demonstrated an insignificant prolonged onset of sensory block. Similar findings were presented in motor block onset by Kathuria. On the other hand, motor block onset was slightly earlier in the magnesium group in the Faiz study, although this difference was insignificant. Motor block recovery was significantly faster in Faiz study when compared to placebo while Kathuria study demonstrated later recovery. Sensory block recovery was reported by Kathuria, and it was also delayed compared to placebo. Duration of surgery was shorter in Faiz compared to placebo, whereas Kathuria showed longer surgery duration, however, these differences were insignificant. Furthermore, VAS pain assessment scores were reported solely in the Kathuria study, with magnesium groups showing a lower level in all intervals except for 24 hours, which was higher compared to placebo. The duration of spinal anesthesia was significantly longer in magnesium groups when compared to placebo as reported by Kathuria. However, a major difference regarding motor block onset definition was presented as Kathuria identified it as Bromage 3 while Faiz decided to accept Bromage 2 as the onset of motor block. This is a possible explanation of the differences between the 2 studies regarding motor block onset and recovery and duration of surgery. Faiz identified the T10 dermatome level as the onset of sensory block while Kathuria accepted the T8-9 dermatome level.^[13,14]^

#### 4.2.2. When compared with neostigmine

Faiz study compared magnesium to neostigmine. Neostigmine showed an earlier onset of sensory block while magnesium had the earliest onset of motor block, however, these findings were insignificant. The time for motor block recovery was significantly longer in the neostigmine group. The neostigmine group had the highest incidence of side effects, specifically nausea and vomiting.^[13]^

Overall, IT administration of MgSO4 is associated with improved outcomes in terms of extending sensory and motor block recovery time. This extension leads to a longer duration for the patient to feel or move his lower limbs, thus increasing the time required for analgesics.

### 4.3. IT vs IV MgSO4 in orthopedic surgery

The principal objective of numerous clinical trials has been to amplify the dynamic recovery of function after orthopedic surgery. These trials have sought to investigate and compare the effects of administering MgSO4 either IV or IT before surgery. Upon review of the literature, 3 clinical trials were identified, all of which investigated the outcomes after administering MgSO4 IT and compared it to IV route. These trials included seventy-five, sixty, and ninety adult patients with an ASA physical status I or II.^[15–17]^

Patients undergoing different orthopedic surgeries under spinal anesthesia, unilateral total hip arthroplasty, elective fixation of a femoral shaft fracture using interlocking fixation, and extracapsular hip fracture surgery, were enrolled and randomized into different groups. One of the trials had 2 phases, experimental and clinical.^[16]^

Samir et al compared the administration of MgSO4 added to bupivacaine and fentanyl IT with IV administration. While the control group received hyperbaric bupivacaine and fentanyl.^[15]^ In contrast, Kumar et al compared IT administration of MgSO4 with bupivacaine and IV administration of MgSO4 with IT bupivacaine. The control group had IT bupivacaine with normal saline.^[17]^ In the clinical phase of Messeha and Boshra study, patients were randomized into 3 groups, the intervention groups received an IT mixture of morphine with bupivacaine and MgSO4 or an IV mixture of morphine and MgSO4 with IT bupivacaine. While the control group received a mixture of morphine with IT bupivacaine.^[16]^ Refer to Table 3 for better understanding.

**Table 3:** Summary of clinical studies comparing intravenous and intrathecal magnesium sulfate in orthopedic surgery.

Study ID	Country	Type of surgery	Intervention	Control	Conclusion
Samir et al 2013^[1]^	Egypt	Total hip arthroplasty	- Group 2: IT^a^ MgSO4 added to bupivacaine and fentanyl (n^b^ = 25) - Group 3: IV^c^ MgSO4 added to bupivacaine and fentanyl (n = 25)	- Group 1: bupivacaine and fentanyl (n = 25)	Postoperative analgesia after total hip replacement improved with: - IV infusion of MgSO4 - IT injection of MgSO4 IV infusion of MgSO4 reduced intraoperative blood loss
Kumar et al 2016^[2]^	India	Extracapsular hip fracture	- Group 2: IV MgSO4 with IT bupivacaine (n = 30) - Group 3: IT MgSO4 with bupivacaine (n = 30)	- Group 1: IT bupivacaine with normal saline (n = 30)	Patients receiving IV MgSO4 experienced: - Longer pain-free intervals - Lower pain scores - Extended sensory and motor blockade - Reduced need for rescue analgesia (*P* < .05)
Messeha and Boshra 2016^[3]^	Egypt	Elective Fixation of a femoral shaft	- Group 2: IT mixture of morphine with bupivacaine and MgSO4 (n = 20) - Group 3: IV mixture of morphine and MgSO4 with IT bupivacaine (n = 20)	- Group 1: a mixture of morphine with IT bupivacaine (n = 20)	Experimental and clinical investigations show: - IV infusion of MgSO4 enhances the analgesic effect of IT morphine - IV infusion achieves a similar degree of enhancement as the IT route

a IT: Intrathecal

b n: number of patients

c IV: Intravenous

1.Samir EM, Badawy SS, Hassan AR. Intrathecal vs intravenous magnesium as an adjuvant to bupivacaine spinal anesthesia for total hip arthroplasty. Egypt J Anaesth. 2013;29(4):395-400. doi:10.1016/j.egja.2013.06.004

2.Kumar A, Chaudhary UK, Kansal D, Rana S, Sharma V, Kumar P. Comparison of intravenous Magnesium Sulfate with intrathecal Magnesium Sulfate for postoperative analgesia in orthopedic patients undergoing extracapsular hip fracture surgery. Int J Basic Clin Pharmacol. 2017;6(1):159-166. doi:10.18203/2319-2003.ijbcp20164773

3.Messeha MM, Boshra V. Comparison of the antinociceptive effect of systemic versus intrathecal magnesium sulfate on spinal morphine analgesia. Magnes Res. 2016;29(1):22-33. doi:10.1684/mrh.2016.0396

**Figure 2.**
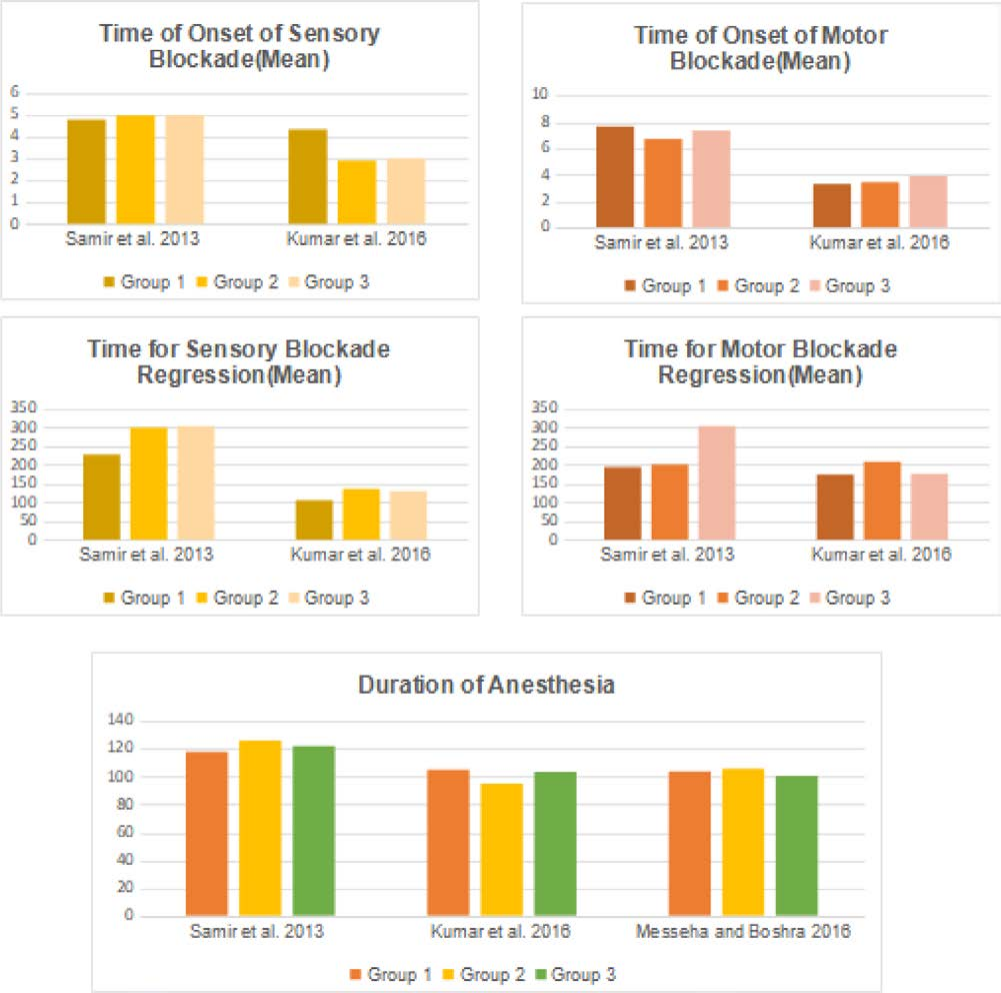
Outcomes comparison between studies comparing intravenous and intrathecal administration of magnesium sulfate.

In Kumar et al, IV MgSO4 group had longer sensory and motor blockade compared to the patients in the IT group or control group.^[17]^ In contrast, Samir et al did not report significant differences between study groups in onset time and maximum sensory level achieved, as well as onset and duration of motor block. Regarding postoperative pain, Samir et al reported similar improvements in postoperative analgesia for both the IV and IT groups. Postoperative pain scores and 24-hour analgesic consumption were lower in intervention groups with insignificant differences between them compared to the control group.^[15]^ However, Kumar et al reported a longer pain-free interval and lower pain scores for IV MgSO4.^[17]^ Figure 2 illustrates the differences in outcomes among the groups across the studies.

Concerning Intraoperative blood loss and postoperative mg levels, Samir et al reported a significant decrease in intraoperative blood loss associated with IV infusion of MgSO4. However, postoperative mg levels were higher in this group, without consequential side effects.^[15]^

Messeha and Boshra study reported that IV MgSO4 was equally effective in potentiating the analgesic effect of morphine compared to the IT route. The role of IV MgSO4 administration was emphasized in avoiding the side effects associated with the IT route, such as motor paralysis.^[16]^

### 4.4. Strengths and limitations

In our comprehensive literature review, we employed strict criteria and a thorough search strategy for the inclusion of studies. After searching major databases, clinical trials registries, and gray literature, our study predominantly comprised randomized clinical trials with minimal risk-of-bias, thereby strengthening the evidence base.

By synthesizing the findings from multiple clinical trials, our review, to our knowledge, is the first to offer a clearer understanding of the effectiveness and safety of IT MgSO4 in orthopedic surgery. Additionally, it highlights gaps and inconsistencies in current research. Clinically, our review lays the groundwork for future research in the field, advocating for further investigations into the potential benefits and risks associated with using IT MgSO4 in orthopedic surgery. Utilizing the Cochrane Quality Assessment Tool (RoB V2) to rigorously assess the methodological quality of the included trials enhances the reliability of our review.

Due to the heterogeneity of the included studies, conducting a formal analysis was not possible. Future systematic reviews should incorporate meta-analysis and compare the findings from each newly published trial. Additionally, due to differences in patient populations, surgical settings, and underrepresentation of certain groups like pediatrics and the elderly, limited generalizability is another constraint of this study.

Furthermore, most included trials did not report on the long-term effects of the intervention, which is a crucial aspect in the field of orthopedic surgery. Therefore, future research should aim to address these limitations.

## 5. Conclusion

MgSO4 is a valuable adjuvant in anesthesia, especially in the realm of orthopedic surgery, significantly enhancing postoperative pain management. While its efficacy is evident, the choice between the mode of administration remains a critical decision. IT administration offers several advantages such as delayed onset and extended block duration. However, the choice between the 2 modes of administration should be individualized, considering patient-specific factors. This review highlights the need for further research to optimize the use of MgSO4 in surgical procedures.

## Acknowledgments

We thank the contribution of Noor Alawad who assisted in designing the figure in this study.

## Author contributions

**Conceptualization:** Lana Sbitan, Noor Alzraikat.

**Data curation:** Lana Sbitan, Ayman Issa Nabhan, Bana Zuhair Alafandi, Omar Alzraikat, Noor Alzraikat.

**Investigation:** Bana Zuhair Alafandi.

**Methodology:** Lana Sbitan.

**Project administration:** Lana Sbitan.

**Resources:** Lana Sbitan, Ayman Issa Nabhan.

**Supervision:** Lana Sbitan, Noor Alzraikat.

**Validation:** Lana Sbitan, Noor Alzraikat.

**Visualization:** Lana Sbitan, Omar Alzraikat.

**Writing – original draft:** Lana Sbitan, Ayman Issa Nabhan, Bana Zuhair Alafandi, Omar Alzraikat, Noor Alzraikat.

**Writing – review & editing:** Lana Sbitan, Ayman Issa Nabhan, Bana Zuhair Alafandi, Omar Alzraikat, Noor Alzraikat.

## Supplementary Material


