# Supplementary material for: Magnesium sulfate for postoperative pain in orthopedic surgery: A narrative review
Source: Medicine (Baltimore). 2024 Jun 14;103(24):e38522. doi: 10.1097/MD.0000000000038522 (PMC11175876; doi:10.1097/MD.0000000000038522)
Supplement: Supplementary file 1 [file medi-103-e38522-s001.docx]

| **Study ID** | **Shukla et al. 2011**[**^1^**](https://www.zotero.org/google-docs/?AVNi1H) | **Choudhury et al. 2016**[**^2^**](https://www.zotero.org/google-docs/?ngZkxg) | **Xiao et al. 2017**[**^3^**](https://www.zotero.org/google-docs/?mpMIoS) | **Sen et al. 2020**[**^4^**](https://www.zotero.org/google-docs/?cFYIOU) | **Faiz et al. 2012**[**^5^**](https://www.zotero.org/google-docs/?NU38iE) | **Kathuria at al. 2014**[**^6^**](https://www.zotero.org/google-docs/?SxuvGs) | **Samir et al. 2013**[**^7^**](https://www.zotero.org/google-docs/?Do5lyW) | **Kumar et al. 2016**[**^8^**](https://www.zotero.org/google-docs/?aKBuaO) | **Messeha and Boshra 2016**[**^9^**](https://www.zotero.org/google-docs/?WcVVm8) |
| --- | --- | --- | --- | --- | --- | --- | --- | --- | --- |
| **Randomization** | Low Risk | Low Risk | Low Risk | Low Risk | Low Risk | Low Risk | Low Risk | Low Risk | Low Risk |
| **Deviations from intended interventions** | Low Risk | Low Risk | Low Risk | Low Risk | Low Risk | Low Risk | Low Risk | Unclear Risk | Unclear Risk |
| **Missing outcome data** | Low Risk | Low Risk | Low Risk | Low Risk | Low Risk | Low Risk | Low Risk | Low Risk | High Risk |
| **Measurement of the outcome** | Low Risk | Low Risk | Low Risk | Unclear Risk | Low Risk | Unclear Risk | Unclear Risk | Low Risk | Unclear Risk |
| **Selection of the reported results** | Low Risk | Low Risk | Low Risk | Low Risk | Low Risk | Low Risk | Low Risk | Unclear Risk | High Risk |
| **Overall Bias** | **Low Risk** | **Low Risk** | **Low Risk** | **Low Risk** | **Low Risk** | **Low Risk** | **Low Risk** | **Unclear Risk** | **High Risk** |

**Supplemental Digital Content: Risk of Bias Assessment of Clinical Trials Using the RoB V2 Quality Assessment Tool**

[1. Shukla D, Verma A, Agarwal A, Pandey HD, Tyagi C. Comparative study of intrathecal dexmedetomidine with intrathecal magnesium sulfate used as adjuvants to bupivacaine. *J Anaesthesiol Clin Pharmacol*. 2011;27(4):495-499. doi:10.4103/0970-9185.86594](https://www.zotero.org/google-docs/?3Wk519)

[2. Choudhury B, Pathak D, Chauhan RC, L C, Mondal D. EFFECT OF INTRATHECAL NALBUPHINE AND MAGNESIUM SULPHATE USED AS ADJUVANTS WITH BUPIVACAINE IN SPINAL ANAESTHESIA FOR LOWER ABDOMINAL SURGERY: A COMPARISON. *J Evol Med Dent Sci*. 2016;5:4922-4926. doi:10.14260/jemds/2016/1118](https://www.zotero.org/google-docs/?3Wk519)

[3. Xiao F, Xu W, Feng Y, et al. Intrathecal magnesium sulfate does not reduce the ED50 of intrathecal hyperbaric bupivacaine for cesarean delivery in healthy parturients: a prospective, double blinded, randomized dose-response trial using the sequential allocation method. *BMC Anesthesiol*. 2017;17:8. doi:10.1186/s12871-017-0300-z](https://www.zotero.org/google-docs/?3Wk519)

[4. Sen J, Singh S, Sen B. The Effect of Intrathecal Magnesium Sulphate on Bupivacaine-Fentanyl Subarachnoid Block for Infraumbilical Surgeries. *J Evol Med Dent Sci*. 2020;9(10):780-785. doi:10.14260/jemds/2020/170](https://www.zotero.org/google-docs/?3Wk519)

[5. Faiz SHR, Rahimzadeh P, Sakhaei M, Imani F, Derakhshan P. Anesthetic effects of adding intrathecal neostigmine or magnesium sulphate to bupivacaine in patients under lower extremities surgeries. *J Res Med Sci Off J Isfahan Univ Med Sci*. 2012;17(10):918-922.](https://www.zotero.org/google-docs/?3Wk519)

[6. Kathuria B, Luthra N, Gupta A, Grewal A, Sood D. Comparative Efficacy of Two Different Dosages of Intrathecal Magnesium Sulphate Supplementation in Subarachnoid Block. *J Clin Diagn Res JCDR*. 2014;8(6):GC01-GC05. doi:10.7860/JCDR/2014/8295.4510](https://www.zotero.org/google-docs/?3Wk519)

[7. Samir EM, Badawy SS, Hassan AR. Intrathecal vs intravenous magnesium as an adjuvant to bupivacaine spinal anesthesia for total hip arthroplasty. *Egypt J Anaesth*. 2013;29(4):395-400. doi:10.1016/j.egja.2013.06.004](https://www.zotero.org/google-docs/?3Wk519)

[8. Kumar A, Chaudhary UK, Kansal D, Rana S, Sharma V, Kumar P. Comparison of intravenous Magnesium Sulphate with intrathecal Magnesium Sulphate for post- operative analgesia in orthopaedic patients undergoing extracapsular hip fracture surgery. *Int J Basic Clin Pharmacol*. 2017;6(1):159-166. doi:10.18203/2319-2003.ijbcp20164773](https://www.zotero.org/google-docs/?3Wk519)

[9. Messeha MM, Boshra V. Comparison of the antinociceptive effect of systemic versus intrathecal magnesium sulphate on spinal morphine analgesia. *Magnes Res*. 2016;29(1):22-33. doi:10.1684/mrh.2016.0396](https://www.zotero.org/google-docs/?3Wk519)
